# "Bovril" Specialties

**Published:** 1911-02-04

**Authors:** 


### "BOVRIL" SPECIALTIES.

### A WALK THROUGH THE OLD STREET BUILDINGS.

On Thursday afternoon last a large number of medical practitioners took advantage of the invitation extended by the directors of Bovril, Limited, to visit the premises of the firm in Old Street, and to inspect the marvellous methods of preparing the various specialities for which the establishment is famous. An invitation to "Bovrils" is not a thing to be lightly thrown away, and everyone who attended on Thursday carried away with him or her pleasant memories. The company gave the visitors a hearty welcome, and the arrangements were exceptionally good when one considers the fact that a very large number of medical men attended.

The visitors were received by the directors, and were introduced by Sir James Crichton-Brown, who is a member of the board, to the Earl of Erroll, who is the chairman. After the formal reception they were shown round the premises, the way being marked out by clearly written notices and a winding Ariadne's thread of red that guided them through this labyrinth of rooms. Not that there were not enough attendants and instructors to show the way and to explain every process and detail to those who were interested in the manufacture of Bovril, Invalid Bovril, Virol, and the many other palatable and nourishing things that the firm has put on the market. Our own attention was particularly attracted to the raw material, as it may be termed, from which Bovril is made. The dried and finely ground beef, which is technically styled "Beef Proteid," was conspicuous in every room. It is a light brownish yellow powder, practically odourless, and with a slightly sweetish taste, and when incorporated with certain salts and other substances it forms the basis of Bovril. In our opinion it is an excellent invalid food, and many medical men would be glad to be able to procure it in its pristine powdered state. We would suggest to the firm that "Beef Proteid" be put up in small tins or packets and placed on the market, so that those who wish to prescribe it to their patients may be able to obtain it. At present there is no reliable scraped beef or powdered meat preparation to be obtained in England, and we throw out this hint for what it is worth. The preparation which the firm has already placed on the market, and in which this Beef Proteid is mixed with a certain amount of fat, is styled "pemmican," and can be obtained only on application to the head offices, since it is specially made for foreign expeditions—many of the most famous of which Bovril has provisioned in the past. Pemmican is a very palatable food, but for invalid use its fat content is too high, and we much prefer therefore the original beef proteid. We are informed that the firm will gladly make up combinations of beef proteid to suit the requirements of special patients, but such combinations can, of course, only be obtained on direct application to the headquarters. What the doctor wants is a really reliable beef powder that can be prescribed on bread and butter, just as nowadays biltong, sliced very thin, is prescribed, or, as in the German clinics, scraped ham is used.

A feature of the day was the excellent series of preparations showing the food values of various articles of diet which was exhibited in one of the rooms. This is a most useful and instructive exhibition, and we trust that the firm will be able to show the preparations to the general public. It is when one sees such collections of truly valuable educational exhibits that one realises what the English public loses through not having a permanent medico-institutional museum of the nature of the Kaiserin Friedrich Haus at Berlin.

The special preparations which are made by the firm were on view in a separate room, and attracted much attention. Many of them are doubtless new even to the medical profession : certainly the compressed vegetables and emergency rations, which can be obtained at the large stores, such as the Army and Navy, appeared to be wholly unknown to many of the guests. The raw Bovril—or beef extract—was shown in tins, as it comes from the various collecting stations in the Argentine and in Australia, and was surreptitiously tasted by many visitors, who expressed the opinion that it was "not at all bad!" Then the packing and bottling rooms were visited, and here the elaborate precautions to ensure cleanliness and exactitude were inspected. In every room there were attractive posters and souvenirs, and in every department a courteous chemist or skilled attendant was ready to give a wealth of information to those anxious to obtain particulars with regard to the manufacture and composition of the various food specialities of Bovril Limited. Finally the visitors entered a large packing-room which had been turned into a special demonstration hall, where the popular Pathé films, showing micro-organisms in full war array and leucocytes in an earnest phacocytic mood, were shown. Then the visitors were ushered into another large room, which had been daintily decorated, and where a champagne luncheon was served.

Bovril is so well known that it is scarcely possible that such excursions to the company's premises will win for it more adherents than it already possesses. But they will at least show medical men the care and the precautions which are taken to ensure that the product that comes to us in the familiar jar or bottle is absolutely trustworthy, and they will also show them that there are many other equally meritorious products for which the firm is responsible. These products—such as the Beef Proteid and Pemmican, already mentioned—are not known so well as they deserve, and those who visited the Bovril headquarters on Thursday must have been grateful for the opportunity of becoming better acquainted with their possibilities.

